# Poly[[bis­{μ_3_-2-[(3,5-dimethyl-1*H*-pyrazol-1-yl)(phen­yl)meth­yl]propane­dioato}tetra­sodium(I)] 7.5-hydrate]

**DOI:** 10.1107/S1600536810028515

**Published:** 2010-07-24

**Authors:** Ihssan Meskini, Maria Daoudi, Jean-Claude Daran, Taibi Ben Hadda, Hafid Zouihri

**Affiliations:** a Laboratoire de Chimie Organique, Faculté des Sciences Dhar el Mahraz, Université Sidi Mohammed Ben Abdellah, Fès, Morocco; b Laboratoire de Chimie de Coordination, 205 Route de Narbonne, 31077 Toulouse Cedex, France; c Laboratoire de Chimie des Matériaux, Université Mohammed 1ier, Oujda, Morocco; d Laboratoires de Diffraction des Rayons X, Division UATRS, Centre National pour la Recherche Scientifique et Technique, Rabat, Morocco

## Abstract

The asymmetric unit of the title polymer, {[Na_4_(C_15_H_14_N_2_O_4_)_2_]·7.5H_2_O}_*n*_, contains two 2-[(3,5-dimethyl-1*H*-pyrazol-1-yl)(phen­yl)meth­yl]propane­dioate (ppmp) anions, eight water mol­ecules (one located on a twofold rotation axis) and five sodium cations (one located on an inversion center and the other one located on a twofold rotation axis). The carboxyl­ate groups of the ppmp anions and the water mol­ecules bridge the Na cations, forming a two-dimensional polymeric structure. In the structure there are two types of coordination environment around the metal cations: one Na cation is coordinated by five O atoms in a distorted square-pyramidal geometry while the other four Na cations are coordinated by six O atoms in a distorted octa­hedral geometry. Extensive O—H⋯O and O—H⋯N hydrogen bonding is present in the crystal structure. The H atoms of one methyl group of the ppmp anion are disordered equally over two positions.

## Related literature

For related compounds displaying biological activity, see: Dayam *et al.* (2007 ▶); Patil *et al.* (2007 ▶); Ramkumar *et al.* (2008 ▶); Sechi *et al.* (2009 ▶); Zeng *et al.* (2008 ▶). For the synthetic procedure, see: Pommier & Neamati (2006 ▶).
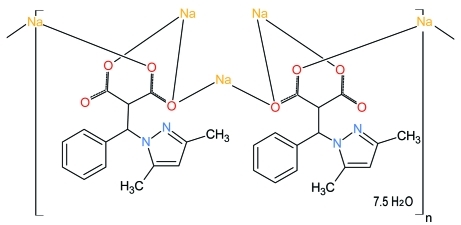


## Experimental

### 

#### Crystal data

[Na_4_(C_15_H_14_N_2_O_4_)_2_]·7.5H_2_O
*M* *_r_* = 799.64
Monoclinic, 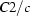

*a* = 31.8211 (11) Å
*b* = 14.4951 (4) Å
*c* = 16.1113 (5) Å
β = 102.139 (3)°
*V* = 7265.2 (4) Å^3^
*Z* = 8
Mo *K*α radiation
μ = 0.16 mm^−1^
*T* = 293 K
0.45 × 0.38 × 0.19 mm

#### Data collection

Bruker X8 APEXII CCD area-detector diffractometer
76380 measured reflections
9034 independent reflections
6490 reflections with *I* > 2σ(*I*)
*R*_int_ = 0.049

#### Refinement

*R*[*F* ^2^ > 2σ(*F* ^2^)] = 0.036
*wR*(*F* ^2^) = 0.130
*S* = 1.02
9034 reflections
488 parameters
H-atom parameters constrained
Δρ_max_ = 0.53 e Å^−3^
Δρ_min_ = −0.44 e Å^−3^

### 

Data collection: *APEX2* (Bruker, 2005 ▶); cell refinement: *SAINT* (Bruker, 2005 ▶); data reduction: *SAINT*; program(s) used to solve structure: *SHELXS97* (Sheldrick, 2008 ▶); program(s) used to refine structure: *SHELXL97* (Sheldrick, 2008 ▶); molecular graphics: *PLATON* (Spek, 2009 ▶); software used to prepare material for publication: *publCIF* (Westrip, 2010 ▶).

## Supplementary Material

Crystal structure: contains datablocks I, global. DOI: 10.1107/S1600536810028515/xu2795sup1.cif

Structure factors: contains datablocks I. DOI: 10.1107/S1600536810028515/xu2795Isup2.hkl

Additional supplementary materials:  crystallographic information; 3D view; checkCIF report

## Figures and Tables

**Table 1:** Selected bond lengths (Å)

Na1—O3	2.3113 (11)
Na1—O11	2.4177 (11)
Na1—O12	2.4028 (13)
Na2—O5^i^	2.3507 (13)
Na2—O5^ii^	2.3417 (13)
Na2—O8	2.2818 (13)
Na2—O21	2.4546 (13)
Na2—O22	2.3253 (13)
Na3—O2	2.4259 (14)
Na3—O31	2.4430 (14)
Na3—O4	2.5862 (13)
Na4—O3^iii^	2.6812 (15)
Na4—O6	2.6565 (14)
Na4—O11^iv^	2.3956 (14)
Na4—O12^iii^	2.4434 (15)
Na4—O41	2.4634 (11)
Na4—O42	2.3423 (15)
Na5—O4	2.3952 (13)
Na5—O5	2.5592 (13)
Na5—O21^v^	2.3176 (13)
Na5—O22^i^	2.4275 (14)
Na5—O31	2.5011 (14)
Na5—O51	2.4478 (14)

Symmetry codes: (i) 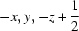; (ii) 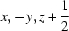; (iii) 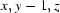; (iv) 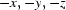; (v) 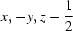.

**Table 2:** Hydrogen-bond geometry (Å, °)

*D*—H⋯*A*	*D*—H	H⋯*A*	*D*⋯*A*	*D*—H⋯*A*
O11—H111⋯O2^i^	0.86	1.97	2.7404 (16)	149
O11—H11*B*⋯O4	0.85	1.88	2.7067 (16)	165
O12—H121⋯O1^i^	0.84	1.90	2.7391 (17)	174
O21—H211⋯O6^i^	0.85	1.92	2.7566 (17)	167
O21—H212⋯O7	0.85	1.97	2.8007 (16)	165
O22—H221⋯O31	0.85	2.10	2.8381 (17)	145
O22—H222⋯O11^i^	0.85	2.31	2.9707 (16)	135
O22—H222⋯O6^ii^	0.85	2.45	3.0532 (18)	128
O31—H31*A*⋯O8	0.85	1.87	2.7128 (16)	169
O31—H31*B*⋯O51	0.84	2.09	2.8085 (17)	142
O41—H411⋯O7	0.84	2.27	3.1061 (16)	172
O42—H421⋯O1^iii^	0.85	1.92	2.7582 (17)	175
O42—H422⋯O7	0.85	2.03	2.8639 (17)	168
O51—H511⋯N4	0.86	1.99	2.8424 (18)	171
O51—H512⋯N2	0.85	1.94	2.7799 (18)	169

Symmetry codes: (i) 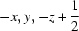; (ii) 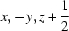; (iii) 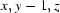.

**Fig. 1.**
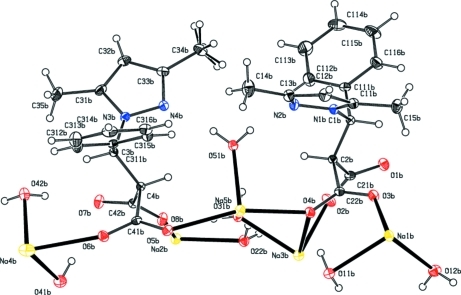
Molecular structure of the title compound with the atom-labelling scheme. Displacement ellipsoids are drawn at the 30% probability level. H atoms are represented as small spheres of arbitrary radii.

**Fig. 2.**
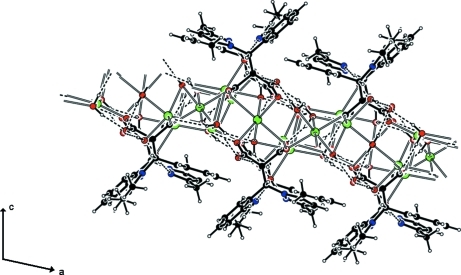
Partial packing view showing the chain generated by C—H···O hydrogen bonds shown as dashed lines.

## supplementary crystallographic information

### Comment

The rational design of new HIV-1 Integrase (H–I) inhibitors, one validated target for chemotherapeutic intervention (Dayam *et al.,* 2007), is fundamentally based on intermolecular coordination between H—I / chemical inhibitor / metals (Mg^+2^ and Mn^+2^, co-factors of the enzyme), leading in the formation of bimetallic complexes (Zeng *et al.,* 2008; Sechi *et al.*, 2009). Thereby, several bimetallic metal complexes, in many cases exploring the known-well polydentate ligands, appear in this scenario as the most promising concept to employ in either enzyme / drug interaction or electron transfer process, in the last case involving the biological oxygen transfer (Sechi *et al.,* 2009; Ramkumar *et al.,* 2008). Another exciting example of application for such polydentate ligand involves the synergic water activation, that occurs *via* the so-called -remote metallic atoms-. Such organometallic compounds are structurally deemed to promote or block the H—I activity (Zeng *et al.,* 2008).

The asymmetric unit of the title polymer contains two (3,5-dimethyl-1H-pyrazol-1-yl)(phenyl)methylpropanedioate (ppmp) anions, eight water molecules (O41 atom located at a twofold rotation axis) and five sodium cations (Na1 located on an inversion center and Na3 located at a twofold rotation axis). The carboxyl groups of ppmp anions and water molecules bridge the Na cations to form the two-dimensional polymeric structure. In the structure there are two types of coodination environment around the metal cations. The Na2 cation is coordinated by five oxygen atoms with a distorted square-pyramidal geometry; the other four Na cations are coordinated by six oxygen atoms with the distorted octahedral geometry. The Na—O bond distances ate ranged from 2.3113 (11) to 2.6812 (15) Å (Table 1). The extensive O—H···O and O—H···N hydrogen bonding is present in the crystal structure (Table 2).

### Experimental

A mixture of the sodium salt of 2-[(phenyl)-3,5-dimethyl-pyrazol-1-yl-]-malonic acid (Pommier & Neamati, 2006) (0.2 g, 0.61 mmol) and (0,13, 1.22 mmol) of sodium dicarbonate in water (5 ml) was stirred at room temperature, then (0.047 g, 0.305 mmol) of (VOSO_4_) was added. The mixture was allowed to stand to ambient temperature. Single crystals suitable for X-ray diffraction were obtained a few days later. Yield: 37%.

### Refinement

All H atoms attached to C were fixed geometrically and treated as riding with C—H = 0.96 Å (methyl), 0.98 Å (methine) or 0.93 Å (aromatic) with *U*_iso_(H) = 1.2*U*_eq_(C) or 1.5*U*_eq_(methyl). H atoms of water molecule were located in difference Fourier maps and included in the subsequent refinement using restraints (O—H= 0.85 (1) Å and H···H = 1.39 (2) Å) with *U*_iso_(H) = 1.5*U*_eq_(O). In the last stage of refinement, they were treated as riding on their parent O atoms. The H atoms of the one methyl group in the ligand are disordered equally over two positions.

### Crystal data

**Table:**

[Na_4_(C_15_H_14_N_2_O_4_)_2_]·7.5H_2_O	*F*(000) = 3352
*M**_r_* = 799.64	*D*_x_ = 1.462 Mg m^−^^3^
Monoclinic, *C*2/*c*	Mo *K*α radiation, λ = 0.71073 Å
Hall symbol: -C 2yc	Cell parameters from 2648 reflections
*a* = 31.8211 (11) Å	θ = 1.5–26.3°
*b* = 14.4951 (4) Å	µ = 0.16 mm^−^^1^
*c* = 16.1113 (5) Å	*T* = 293 K
β = 102.139 (3)°	Block, colourless
*V* = 7265.2 (4) Å^3^	0.45 × 0.38 × 0.19 mm
*Z* = 8	

### Data collection

**Table:**

Bruker X8 APEXII CCD area-detector diffractometer	6490 reflections with *I* > 2σ(*I*)
Radiation source: fine-focus sealed tube	*R*_int_ = 0.049
graphite	θ_max_ = 28.3°, θ_min_ = 1.3°
φ and ω scans	*h* = −42→42
76380 measured reflections	*k* = −19→19
9034 independent reflections	*l* = −21→21

### Refinement

**Table:**

Refinement on *F*^2^	Primary atom site location: structure-invariant direct methods
Least-squares matrix: full	Secondary atom site location: difference Fourier map
*R*[*F*^2^ > 2σ(*F*^2^)] = 0.036	Hydrogen site location: inferred from neighbouring sites
*wR*(*F*^2^) = 0.130	H-atom parameters constrained
*S* = 1.02	*w* = 1/[σ^2^(*F*_o_^2^) + (0.0783*P*)^2^ + 2.736*P*] where *P* = (*F*_o_^2^ + 2*F*_c_^2^)/3
9034 reflections	(Δ/σ)_max_ = 0.006
488 parameters	Δρ_max_ = 0.53 e Å^−^^3^
0 restraints	Δρ_min_ = −0.44 e Å^−^^3^

### Special details

**Table:**

Experimental. IR (KBr, [UTF-8]I^1/2^ cm-1): 1592.46 (C=O), 3248 (OH).The data collection nominally covered a sphere of reciprocal space, by a combination of seven sets of exposures; each set had a different φ angle for the crystal and each exposure covered 0.5° in ω and 20 s in time. The crystal-to-detector distance was 37.5 mm.
Geometry. All e.s.d.'s (except the e.s.d. in the dihedral angle between two l.s. planes) are estimated using the full covariance matrix. The cell e.s.d.'s are taken into account individually in the estimation of e.s.d.'s in distances, angles and torsion angles; correlations between e.s.d.'s in cell parameters are only used when they are defined by crystal symmetry. An approximate (isotropic) treatment of cell e.s.d.'s is used for estimating e.s.d.'s involving l.s. planes.
Refinement. Refinement of *F*^2^ against ALL reflections. The weighted *R*-factor *wR* and goodness of fit *S* are based on *F*^2^, conventional *R*-factors *R* are based on *F*, with *F* set to zero for negative *F*^2^. The threshold expression of *F*^2^ > σ(*F*^2^) is used only for calculating *R*-factors(gt) *etc*. and is not relevant to the choice of reflections for refinement. *R*-factors based on *F*^2^ are statistically about twice as large as those based on *F*, and *R*- factors based on ALL data will be even larger.

### Fractional atomic coordinates and isotropic or equivalent isotropic displacement parameters (Å^2^)

**Table:**

	*x*	*y*	*z*	*U*_iso_*/*U*_eq_	Occ. (<1)
C1	0.13673 (5)	0.43818 (11)	0.21255 (10)	0.0157 (3)	
H1	0.1288	0.5033	0.2036	0.019*	
C2	0.09753 (5)	0.38687 (10)	0.23022 (10)	0.0146 (3)	
H2	0.1049	0.3215	0.2390	0.018*	
N1	0.14699 (4)	0.40316 (9)	0.13308 (8)	0.0171 (3)	
N2	0.15635 (5)	0.31192 (9)	0.12560 (9)	0.0217 (3)	
C11	0.14801 (5)	0.45027 (12)	0.06112 (11)	0.0211 (3)	
C111	0.17568 (5)	0.43350 (12)	0.28481 (10)	0.0189 (3)	
C112	0.18973 (7)	0.35192 (14)	0.32677 (13)	0.0351 (5)	
H112	0.1747	0.2975	0.3109	0.042*	
C113	0.22592 (7)	0.35053 (16)	0.39193 (15)	0.0451 (6)	
H113	0.2350	0.2953	0.4193	0.054*	
C114	0.24851 (6)	0.43032 (17)	0.41628 (14)	0.0420 (5)	
H114	0.2728	0.4292	0.4599	0.050*	
C115	0.23495 (7)	0.51153 (16)	0.37580 (16)	0.0450 (6)	
H115	0.2501	0.5657	0.3920	0.054*	
C116	0.19862 (6)	0.51313 (14)	0.31076 (14)	0.0328 (4)	
H116	0.1895	0.5687	0.2842	0.039*	
C12	0.15814 (6)	0.38751 (13)	0.00471 (11)	0.0268 (4)	
H12	0.1610	0.3990	−0.0506	0.032*	
C13	0.16321 (6)	0.30294 (12)	0.04696 (12)	0.0249 (4)	
C14	0.17451 (8)	0.21067 (14)	0.01556 (14)	0.0417 (5)	
H14A	0.1753	0.1653	0.0593	0.063*	
H14B	0.2022	0.2141	0.0009	0.063*	
H14C	0.1533	0.1935	−0.0337	0.063*	
C15	0.13940 (7)	0.55134 (13)	0.05108 (13)	0.0308 (4)	
H15A	0.1111	0.5643	0.0598	0.046*	
H15B	0.1414	0.5701	−0.0051	0.046*	
H15C	0.1602	0.5846	0.0920	0.046*	
C21	0.08148 (5)	0.42241 (11)	0.30808 (10)	0.0177 (3)	
C22	0.05932 (5)	0.39513 (11)	0.15432 (10)	0.0163 (3)	
O1	0.09050 (4)	0.50303 (8)	0.33362 (8)	0.0245 (3)	
O2	0.05884 (4)	0.36630 (9)	0.33884 (8)	0.0292 (3)	
O3	0.04974 (4)	0.47344 (8)	0.12450 (8)	0.0246 (3)	
O4	0.03947 (4)	0.32106 (8)	0.12934 (8)	0.0227 (3)	
C3	0.12948 (5)	−0.12031 (11)	0.19213 (10)	0.0152 (3)	
H3	0.1257	−0.1848	0.2073	0.018*	
C4	0.08996 (5)	−0.06637 (11)	0.20531 (10)	0.0141 (3)	
H4	0.0968	−0.0005	0.2054	0.017*	
N3	0.16800 (4)	−0.08512 (9)	0.25020 (9)	0.0177 (3)	
N4	0.17591 (4)	0.00742 (9)	0.25847 (9)	0.0203 (3)	
C31	0.20142 (6)	−0.13530 (13)	0.29413 (12)	0.0249 (4)	
C311	0.13456 (5)	−0.11851 (11)	0.10044 (10)	0.0175 (3)	
C312	0.13502 (6)	−0.20186 (12)	0.05790 (12)	0.0274 (4)	
H312	0.1330	−0.2570	0.0863	0.033*	
C313	0.13850 (7)	−0.20368 (14)	−0.02656 (13)	0.0345 (5)	
H313	0.1389	−0.2598	−0.0543	0.041*	
C314	0.14133 (6)	−0.12223 (14)	−0.06929 (12)	0.0296 (4)	
H314	0.1435	−0.1234	−0.1260	0.035*	
C315	0.14097 (6)	−0.03861 (13)	−0.02781 (11)	0.0255 (4)	
H315	0.1431	0.0163	−0.0565	0.031*	
C316	0.13747 (5)	−0.03674 (12)	0.05677 (11)	0.0222 (3)	
H316	0.1371	0.0195	0.0843	0.027*	
C32	0.23146 (6)	−0.07307 (13)	0.33379 (13)	0.0299 (4)	
H32	0.2578	−0.0862	0.3695	0.036*	
C33	0.21456 (5)	0.01393 (12)	0.30977 (12)	0.0243 (4)	
C34	0.23424 (6)	0.10641 (14)	0.33425 (14)	0.0369 (5)	
H34A	0.2150	0.1539	0.3078	0.055*	0.50
H34B	0.2610	0.1113	0.3159	0.055*	0.50
H34C	0.2394	0.1133	0.3948	0.055*	0.50
H34D	0.2619	0.0984	0.3712	0.055*	0.50
H34E	0.2159	0.1411	0.3631	0.055*	0.50
H34F	0.2375	0.1391	0.2842	0.055*	0.50
C35	0.20217 (7)	−0.23784 (14)	0.29361 (15)	0.0399 (5)	
H35A	0.1782	−0.2610	0.3149	0.060*	
H35B	0.2285	−0.2593	0.3290	0.060*	
H35C	0.2003	−0.2594	0.2366	0.060*	
C41	0.05116 (5)	−0.08420 (11)	0.13273 (10)	0.0154 (3)	
C42	0.07637 (5)	−0.08894 (11)	0.28921 (10)	0.0156 (3)	
O5	0.03524 (4)	−0.01421 (8)	0.09023 (7)	0.0191 (2)	
O6	0.03787 (4)	−0.16533 (8)	0.11998 (8)	0.0230 (3)	
O7	0.08345 (4)	−0.16799 (8)	0.32102 (8)	0.0239 (3)	
O8	0.05735 (4)	−0.02532 (8)	0.31923 (7)	0.0229 (3)	
Na1	0.0000	0.5000	0.0000	0.0202 (2)	
Na2	0.03995 (2)	0.00005 (4)	0.44768 (4)	0.01928 (15)	
Na3	0.0000	0.28300 (6)	0.2500	0.0230 (2)	
Na4	0.03267 (3)	−0.34679 (6)	0.13857 (5)	0.0347 (2)	
Na5	0.04893 (2)	0.15926 (4)	0.10917 (4)	0.02081 (15)	
O11	−0.03287 (4)	0.35116 (8)	0.00998 (7)	0.0201 (2)	
H111	−0.0480	0.3692	0.0450	0.030*	
H11B	−0.0086	0.3365	0.0400	0.030*	
O12	−0.03957 (4)	0.59680 (9)	0.07746 (8)	0.0276 (3)	
H121	−0.0542	0.5642	0.1043	0.041*	
H122	−0.0554	0.6369	0.0489	0.041*	
O21	0.04694 (4)	−0.16789 (8)	0.46475 (7)	0.0231 (3)	
H211	0.0208	−0.1762	0.4404	0.035*	
H212	0.0611	−0.1749	0.4263	0.035*	
O22	0.02907 (4)	0.15783 (8)	0.42770 (8)	0.0231 (3)	
H221	0.0435	0.1722	0.3908	0.035*	
H222	0.0368	0.1942	0.4697	0.035*	
O31	0.04933 (4)	0.15204 (8)	0.26444 (8)	0.0225 (3)	
H31A	0.0500	0.0942	0.2749	0.034*	
H31B	0.0750	0.1673	0.2648	0.034*	
O41	0.0000	−0.27849 (13)	0.2500	0.0307 (4)	
H411	0.0213	−0.2470	0.2736	0.046*	
O42	0.09668 (4)	−0.33909 (8)	0.24137 (8)	0.0279 (3)	
H421	0.0932	−0.3870	0.2691	0.042*	
H422	0.0940	−0.2925	0.2718	0.042*	
O51	0.12258 (4)	0.15927 (8)	0.19318 (8)	0.0242 (3)	
H511	0.1410	0.1169	0.2115	0.036*	
H512	0.1361	0.2027	0.1744	0.036*	

### Atomic displacement parameters (Å^2^)

**Table:**

	*U*^11^	*U*^22^	*U*^33^	*U*^12^	*U*^13^	*U*^23^
C1	0.0160 (7)	0.0154 (7)	0.0155 (8)	−0.0014 (6)	0.0030 (6)	−0.0002 (6)
C2	0.0146 (7)	0.0150 (7)	0.0137 (7)	−0.0007 (5)	0.0018 (6)	0.0003 (6)
N1	0.0197 (7)	0.0161 (6)	0.0162 (7)	−0.0001 (5)	0.0056 (5)	0.0010 (5)
N2	0.0266 (7)	0.0171 (7)	0.0229 (8)	0.0015 (6)	0.0086 (6)	−0.0003 (6)
C11	0.0212 (8)	0.0232 (9)	0.0191 (8)	0.0007 (6)	0.0051 (7)	0.0050 (7)
C111	0.0150 (7)	0.0245 (8)	0.0174 (8)	−0.0013 (6)	0.0039 (6)	−0.0019 (6)
C112	0.0344 (11)	0.0277 (10)	0.0349 (11)	−0.0031 (8)	−0.0113 (9)	0.0033 (8)
C113	0.0409 (12)	0.0434 (13)	0.0409 (13)	0.0032 (10)	−0.0145 (10)	0.0094 (10)
C114	0.0238 (10)	0.0651 (15)	0.0314 (12)	−0.0069 (10)	−0.0074 (8)	−0.0027 (10)
C115	0.0347 (11)	0.0490 (14)	0.0448 (14)	−0.0203 (10)	−0.0066 (10)	−0.0067 (11)
C116	0.0308 (10)	0.0290 (10)	0.0352 (11)	−0.0083 (8)	−0.0006 (8)	0.0001 (8)
C12	0.0344 (10)	0.0299 (10)	0.0178 (9)	0.0019 (7)	0.0090 (7)	0.0022 (7)
C13	0.0286 (9)	0.0248 (9)	0.0234 (9)	−0.0002 (7)	0.0099 (7)	−0.0022 (7)
C14	0.0624 (15)	0.0312 (11)	0.0370 (12)	0.0035 (10)	0.0229 (11)	−0.0055 (9)
C15	0.0402 (11)	0.0237 (9)	0.0294 (10)	0.0042 (8)	0.0093 (8)	0.0096 (8)
C21	0.0153 (7)	0.0232 (8)	0.0133 (8)	−0.0004 (6)	0.0004 (6)	−0.0002 (6)
C22	0.0152 (7)	0.0207 (8)	0.0136 (7)	−0.0004 (6)	0.0044 (6)	−0.0012 (6)
O1	0.0314 (7)	0.0219 (6)	0.0219 (6)	−0.0028 (5)	0.0097 (5)	−0.0056 (5)
O2	0.0354 (7)	0.0332 (7)	0.0227 (7)	−0.0136 (6)	0.0143 (6)	−0.0036 (5)
O3	0.0253 (6)	0.0210 (6)	0.0235 (7)	−0.0020 (5)	−0.0040 (5)	0.0064 (5)
O4	0.0211 (6)	0.0196 (6)	0.0237 (6)	−0.0021 (5)	−0.0035 (5)	−0.0039 (5)
C3	0.0161 (7)	0.0152 (7)	0.0144 (7)	0.0005 (6)	0.0034 (6)	−0.0009 (6)
C4	0.0147 (7)	0.0145 (7)	0.0132 (7)	0.0006 (5)	0.0031 (6)	0.0006 (6)
N3	0.0169 (6)	0.0181 (7)	0.0174 (7)	0.0021 (5)	0.0020 (5)	0.0003 (5)
N4	0.0195 (7)	0.0176 (7)	0.0232 (8)	−0.0007 (5)	0.0031 (6)	−0.0005 (6)
C31	0.0201 (8)	0.0272 (9)	0.0264 (9)	0.0069 (7)	0.0024 (7)	0.0052 (7)
C311	0.0146 (7)	0.0219 (8)	0.0167 (8)	0.0012 (6)	0.0053 (6)	−0.0013 (6)
C312	0.0383 (10)	0.0216 (9)	0.0245 (9)	0.0015 (7)	0.0113 (8)	−0.0018 (7)
C313	0.0460 (12)	0.0328 (11)	0.0262 (10)	0.0016 (9)	0.0110 (9)	−0.0115 (8)
C314	0.0245 (9)	0.0476 (12)	0.0175 (9)	−0.0009 (8)	0.0066 (7)	−0.0037 (8)
C315	0.0236 (9)	0.0345 (10)	0.0184 (9)	−0.0029 (7)	0.0044 (7)	0.0041 (7)
C316	0.0243 (8)	0.0232 (9)	0.0198 (9)	0.0001 (7)	0.0059 (7)	−0.0007 (7)
C32	0.0182 (8)	0.0349 (11)	0.0327 (11)	0.0033 (7)	−0.0033 (7)	0.0026 (8)
C33	0.0180 (8)	0.0295 (10)	0.0251 (9)	−0.0021 (7)	0.0038 (7)	−0.0013 (7)
C34	0.0271 (10)	0.0353 (11)	0.0457 (13)	−0.0096 (8)	0.0022 (9)	−0.0067 (9)
C35	0.0343 (11)	0.0265 (10)	0.0536 (14)	0.0121 (8)	−0.0028 (10)	0.0041 (9)
C41	0.0142 (7)	0.0218 (8)	0.0114 (7)	0.0017 (6)	0.0056 (6)	−0.0003 (6)
C42	0.0168 (7)	0.0176 (7)	0.0118 (7)	−0.0008 (6)	0.0016 (6)	−0.0007 (6)
O5	0.0207 (6)	0.0224 (6)	0.0138 (6)	0.0041 (4)	0.0025 (4)	0.0036 (4)
O6	0.0229 (6)	0.0218 (6)	0.0219 (6)	−0.0030 (5)	−0.0007 (5)	−0.0010 (5)
O7	0.0366 (7)	0.0202 (6)	0.0177 (6)	0.0055 (5)	0.0118 (5)	0.0047 (5)
O8	0.0326 (7)	0.0204 (6)	0.0185 (6)	0.0062 (5)	0.0119 (5)	0.0012 (5)
Na1	0.0243 (5)	0.0168 (4)	0.0175 (5)	−0.0013 (3)	−0.0003 (4)	0.0014 (4)
Na2	0.0231 (3)	0.0201 (3)	0.0154 (3)	0.0022 (2)	0.0059 (3)	−0.0001 (2)
Na3	0.0219 (5)	0.0212 (5)	0.0263 (5)	0.000	0.0060 (4)	0.000
Na4	0.0301 (4)	0.0529 (5)	0.0206 (4)	−0.0048 (3)	0.0045 (3)	−0.0084 (3)
Na5	0.0230 (3)	0.0220 (3)	0.0168 (3)	0.0017 (3)	0.0027 (3)	−0.0014 (3)
O11	0.0199 (6)	0.0241 (6)	0.0160 (6)	0.0008 (4)	0.0033 (5)	0.0002 (5)
O12	0.0253 (6)	0.0326 (7)	0.0259 (7)	0.0014 (5)	0.0080 (5)	0.0062 (5)
O21	0.0230 (6)	0.0302 (7)	0.0165 (6)	0.0021 (5)	0.0049 (5)	0.0016 (5)
O22	0.0264 (6)	0.0201 (6)	0.0236 (6)	0.0001 (5)	0.0073 (5)	−0.0035 (5)
O31	0.0241 (6)	0.0185 (6)	0.0260 (7)	0.0012 (5)	0.0078 (5)	0.0035 (5)
O41	0.0288 (10)	0.0320 (10)	0.0328 (11)	0.000	0.0099 (8)	0.000
O42	0.0353 (7)	0.0197 (6)	0.0292 (7)	−0.0003 (5)	0.0080 (6)	0.0001 (5)
O51	0.0214 (6)	0.0181 (6)	0.0337 (7)	0.0005 (5)	0.0073 (5)	0.0057 (5)

### Geometric parameters (Å, °)

**Table:**

Na1—O3	2.3113 (11)	C13—C14	1.500 (3)
Na1—O3^i^	2.3113 (11)	C14—H14A	0.9600
Na1—O11	2.4177 (11)	C14—H14B	0.9600
Na1—O11^i^	2.4177 (11)	C14—H14C	0.9600
Na1—O12	2.4028 (13)	C15—H15A	0.9600
Na1—O12^i^	2.4028 (13)	C15—H15B	0.9600
Na2—O5^ii^	2.3507 (13)	C15—H15C	0.9600
Na2—O5^iii^	2.3417 (13)	C21—O1	1.252 (2)
Na2—O8	2.2818 (13)	C21—O2	1.256 (2)
Na2—O21	2.4546 (13)	C22—O3	1.245 (2)
Na2—O22	2.3253 (13)	C22—O4	1.2676 (19)
Na3—O2	2.4259 (14)	C3—N3	1.468 (2)
Na3—O2^ii^	2.4259 (14)	C3—C311	1.520 (2)
Na3—O31	2.4430 (14)	C3—C4	1.533 (2)
Na3—O31^ii^	2.4430 (14)	C3—H3	0.9800
Na3—O4	2.5862 (13)	C4—C41	1.533 (2)
Na3—O4^ii^	2.5862 (13)	C4—C42	1.538 (2)
Na4—O3^iv^	2.6812 (15)	C4—H4	0.9800
Na4—O6	2.6565 (14)	N3—C31	1.358 (2)
Na4—O11^v^	2.3956 (14)	N3—N4	1.3661 (19)
Na4—O12^iv^	2.4434 (15)	N4—C33	1.333 (2)
Na4—O41	2.4634 (11)	C31—C32	1.371 (3)
Na4—O42	2.3423 (15)	C31—C35	1.487 (3)
Na5—O4	2.3952 (13)	C311—C312	1.391 (2)
Na5—O5	2.5592 (13)	C311—C316	1.391 (2)
Na5—O21^vi^	2.3176 (13)	C312—C313	1.389 (3)
Na5—O22^ii^	2.4275 (14)	C312—H312	0.9300
Na5—O31	2.5011 (14)	C313—C314	1.379 (3)
Na5—O51	2.4478 (14)	C313—H313	0.9300
Na1—Na4^vii^	3.1655 (8)	C314—C315	1.385 (3)
Na1—Na4^v^	3.1655 (8)	C314—H314	0.9300
Na1—H111	2.6295	C315—C316	1.391 (2)
Na1—H11B	2.4858	C315—H315	0.9300
Na2—Na2^viii^	3.3331 (13)	C316—H316	0.9300
Na2—Na5^iii^	3.4453 (9)	C32—C33	1.393 (3)
Na2—Na5^ii^	3.6158 (9)	C32—H32	0.9300
Na2—H211	2.6231	C33—C34	1.497 (3)
Na2—H212	2.6649	C34—H34A	0.9600
Na2—H221	2.6683	C34—H34B	0.9600
Na3—Na5	3.5036 (8)	C34—H34C	0.9600
Na3—Na5^ii^	3.5036 (8)	C34—H34D	0.9600
Na4—Na1^iv^	3.1655 (8)	C34—H34E	0.9600
Na4—H421	2.6015	C34—H34F	0.9600
Na5—Na2^vi^	3.4453 (9)	C35—H35A	0.9600
Na5—Na2^ii^	3.6158 (9)	C35—H35B	0.9600
Na5—H31B	2.4723	C35—H35C	0.9600
C1—N1	1.476 (2)	C41—O6	1.252 (2)
C1—C111	1.513 (2)	C41—O5	1.2684 (19)
C1—C2	1.530 (2)	C42—O8	1.2544 (19)
C1—H1	0.9800	C42—O7	1.2562 (19)
C2—C22	1.537 (2)	O11—Na4^v^	2.3956 (14)
C2—C21	1.539 (2)	O11—H111	0.8553
C2—H2	0.9800	O11—H11B	0.8487
N1—C11	1.352 (2)	O12—Na4^vii^	2.4434 (15)
N1—N2	1.3665 (19)	O12—H121	0.8438
N2—C13	1.337 (2)	O12—H122	0.8399
C11—C12	1.371 (2)	O21—Na5^iii^	2.3176 (13)
C11—C15	1.493 (2)	O21—H211	0.8507
C111—C116	1.383 (2)	O21—H212	0.8458
C111—C112	1.389 (3)	O22—Na5^ii^	2.4275 (14)
C112—C113	1.386 (3)	O22—H221	0.8489
C112—H112	0.9300	O22—H222	0.8522
C113—C114	1.375 (3)	O31—H31A	0.8540
C113—H113	0.9300	O31—H31B	0.8440
C114—C115	1.371 (3)	O41—Na4^ii^	2.4634 (11)
C114—H114	0.9300	O41—H411	0.8398
C115—C116	1.388 (3)	O42—H421	0.8453
C115—H115	0.9300	O42—H422	0.8494
C116—H116	0.9300	O51—H511	0.8572
C12—C13	1.395 (3)	O51—H512	0.8533
C12—H12	0.9300		
			
N1—C1—C111	110.93 (13)	H111—Na1—H11B	30.6
N1—C1—C2	109.38 (12)	O8—Na2—O22	95.19 (5)
C111—C1—C2	113.78 (13)	O8—Na2—O5^iii^	168.93 (5)
N1—C1—H1	107.5	O22—Na2—O5^iii^	90.64 (5)
C111—C1—H1	107.5	O8—Na2—O5^ii^	100.26 (5)
C2—C1—H1	107.5	O22—Na2—O5^ii^	86.37 (5)
C1—C2—C22	110.71 (12)	O5^iii^—Na2—O5^ii^	89.48 (5)
C1—C2—C21	114.35 (13)	O8—Na2—O21	84.74 (4)
C22—C2—C21	106.25 (12)	O22—Na2—O21	176.60 (5)
C1—C2—H2	108.5	O5^iii^—Na2—O21	90.00 (4)
C22—C2—H2	108.5	O5^ii^—Na2—O21	90.30 (4)
C21—C2—H2	108.5	O8—Na2—Na2^viii^	144.59 (5)
C11—N1—N2	111.61 (13)	O22—Na2—Na2^viii^	87.89 (4)
C11—N1—C1	128.29 (14)	O5^iii^—Na2—Na2^viii^	44.85 (3)
N2—N1—C1	120.09 (12)	O5^ii^—Na2—Na2^viii^	44.63 (3)
C13—N2—N1	105.07 (13)	O21—Na2—Na2^viii^	90.21 (4)
N1—C11—C12	106.54 (15)	O8—Na2—Na5^iii^	125.41 (4)
N1—C11—C15	123.48 (15)	O22—Na2—Na5^iii^	138.59 (4)
C12—C11—C15	129.98 (16)	O5^iii^—Na2—Na5^iii^	47.97 (3)
C116—C111—C112	117.92 (17)	O5^ii^—Na2—Na5^iii^	93.18 (4)
C116—C111—C1	119.36 (16)	O21—Na2—Na5^iii^	42.23 (3)
C112—C111—C1	122.72 (15)	Na2^viii^—Na2—Na5^iii^	64.45 (2)
C113—C112—C111	120.82 (18)	O8—Na2—Na5^ii^	101.49 (4)
C113—C112—H112	119.6	O22—Na2—Na5^ii^	41.53 (3)
C111—C112—H112	119.6	O5^iii^—Na2—Na5^ii^	89.11 (4)
C114—C113—C112	120.4 (2)	O5^ii^—Na2—Na5^ii^	44.86 (3)
C114—C113—H113	119.8	O21—Na2—Na5^ii^	135.15 (4)
C112—C113—H113	119.8	Na2^viii^—Na2—Na5^ii^	59.28 (2)
C115—C114—C113	119.57 (19)	Na5^iii^—Na2—Na5^ii^	123.73 (2)
C115—C114—H114	120.2	O8—Na2—H211	84.3
C113—C114—H114	120.2	O22—Na2—H211	157.7
C114—C115—C116	120.17 (19)	O5^iii^—Na2—H211	93.8
C114—C115—H115	119.9	O5^ii^—Na2—H211	71.8
C116—C115—H115	119.9	O21—Na2—H211	18.9
C111—C116—C115	121.16 (19)	Na2^viii^—Na2—H211	80.0
C111—C116—H116	119.4	Na5^iii^—Na2—H211	50.4
C115—C116—H116	119.4	Na5^ii^—Na2—H211	116.6
C11—C12—C13	106.19 (16)	O8—Na2—H212	67.3
C11—C12—H12	126.9	O22—Na2—H212	162.3
C13—C12—H12	126.9	O5^iii^—Na2—H212	106.3
N2—C13—C12	110.59 (15)	O5^ii^—Na2—H212	98.9
N2—C13—C14	120.33 (16)	O21—Na2—H212	18.4
C12—C13—C14	129.07 (17)	Na2^viii^—Na2—H212	107.8
C13—C14—H14A	109.5	Na5^iii^—Na2—H212	58.4
C13—C14—H14B	109.5	Na5^ii^—Na2—H212	141.3
H14A—C14—H14B	109.5	H211—Na2—H212	29.6
C13—C14—H14C	109.5	O8—Na2—H221	78.7
H14A—C14—H14C	109.5	O22—Na2—H221	17.9
H14B—C14—H14C	109.5	O5^iii^—Na2—H221	105.7
C11—C15—H15A	109.5	O5^ii^—Na2—H221	96.1
C11—C15—H15B	109.5	O21—Na2—H221	163.0
H15A—C15—H15B	109.5	Na2^viii^—Na2—H221	105.4
C11—C15—H15C	109.5	Na5^iii^—Na2—H221	152.0
H15A—C15—H15C	109.5	Na5^ii^—Na2—H221	53.4
H15B—C15—H15C	109.5	H211—Na2—H221	157.1
O1—C21—O2	125.86 (15)	H212—Na2—H221	144.6
O1—C21—C2	119.19 (14)	O2—Na3—O2^ii^	120.30 (8)
O2—C21—C2	114.91 (14)	O2—Na3—O31^ii^	147.53 (5)
O3—C22—O4	125.76 (15)	O2^ii^—Na3—O31^ii^	85.97 (4)
O3—C22—C2	117.81 (14)	O2—Na3—O31	85.97 (4)
O4—C22—C2	116.40 (14)	O2^ii^—Na3—O31	147.53 (5)
C21—O2—Na3	121.68 (11)	O31^ii^—Na3—O31	78.02 (6)
C22—O3—Na1	123.65 (10)	O2—Na3—O4	84.31 (4)
C22—O3—Na4^vii^	152.98 (11)	O2^ii^—Na3—O4	83.51 (4)
Na1—O3—Na4^vii^	78.32 (4)	O31^ii^—Na3—O4	119.87 (4)
C22—O4—Na5	143.51 (11)	O31—Na3—O4	80.41 (4)
C22—O4—Na3	103.32 (10)	O2—Na3—O4^ii^	83.51 (4)
Na5—O4—Na3	89.30 (5)	O2^ii^—Na3—O4^ii^	84.31 (4)
N3—C3—C311	111.60 (12)	O31^ii^—Na3—O4^ii^	80.41 (4)
N3—C3—C4	109.50 (12)	O31—Na3—O4^ii^	119.87 (4)
C311—C3—C4	112.58 (13)	O4—Na3—O4^ii^	155.37 (7)
N3—C3—H3	107.6	O2—Na3—Na5	104.12 (3)
C311—C3—H3	107.6	O2^ii^—Na3—Na5	105.40 (3)
C4—C3—H3	107.6	O31^ii^—Na3—Na5	84.53 (4)
C3—C4—C41	111.15 (12)	O31—Na3—Na5	45.55 (3)
C3—C4—C42	113.80 (12)	O4—Na3—Na5	43.13 (3)
C41—C4—C42	107.72 (12)	O4^ii^—Na3—Na5	161.48 (4)
C3—C4—H4	108.0	O2—Na3—Na5^ii^	105.40 (3)
C41—C4—H4	108.0	O2^ii^—Na3—Na5^ii^	104.12 (3)
C42—C4—H4	108.0	O31^ii^—Na3—Na5^ii^	45.55 (3)
C31—N3—N4	111.53 (14)	O31—Na3—Na5^ii^	84.53 (4)
C31—N3—C3	127.11 (14)	O4—Na3—Na5^ii^	161.48 (4)
N4—N3—C3	121.02 (13)	O4^ii^—Na3—Na5^ii^	43.13 (3)
C33—N4—N3	104.89 (13)	Na5—Na3—Na5^ii^	118.41 (3)
N3—C31—C32	106.47 (16)	O42—Na4—O11^v^	121.53 (5)
N3—C31—C35	122.97 (17)	O42—Na4—O12^iv^	154.89 (6)
C32—C31—C35	130.56 (17)	O11^v^—Na4—O12^iv^	77.79 (5)
C312—C311—C316	118.83 (16)	O42—Na4—O41	84.44 (4)
C312—C311—C3	118.58 (15)	O11^v^—Na4—O41	145.99 (5)
C316—C311—C3	122.56 (14)	O12^iv^—Na4—O41	85.57 (4)
C313—C312—C311	120.71 (17)	O42—Na4—O6	88.10 (5)
C313—C312—H312	119.6	O11^v^—Na4—O6	84.27 (5)
C311—C312—H312	119.6	O12^iv^—Na4—O6	111.20 (5)
C314—C313—C312	120.00 (18)	O41—Na4—O6	74.34 (5)
C314—C313—H313	120.0	O42—Na4—O3^iv^	86.82 (5)
C312—C313—H313	120.0	O11^v^—Na4—O3^iv^	81.18 (4)
C313—C314—C315	120.01 (17)	O12^iv^—Na4—O3^iv^	80.27 (5)
C313—C314—H314	120.0	O41—Na4—O3^iv^	125.19 (6)
C315—C314—H314	120.0	O6—Na4—O3^iv^	159.10 (5)
C314—C315—C316	120.01 (17)	O42—Na4—Na1^iv^	129.79 (4)
C314—C315—H315	120.0	O11^v^—Na4—Na1^iv^	49.18 (3)
C316—C315—H315	120.0	O12^iv^—Na4—Na1^iv^	48.66 (3)
C315—C316—C311	120.43 (16)	O41—Na4—Na1^iv^	132.04 (4)
C315—C316—H316	119.8	O6—Na4—Na1^iv^	129.35 (4)
C311—C316—H316	119.8	O3^iv^—Na4—Na1^iv^	45.64 (3)
C31—C32—C33	105.99 (16)	O42—Na4—H421	18.8
C31—C32—H32	127.0	O11^v^—Na4—H421	130.1
C33—C32—H32	127.0	O12^iv^—Na4—H421	136.4
N4—C33—C32	111.11 (15)	O41—Na4—H421	81.7
N4—C33—C34	120.47 (16)	O6—Na4—H421	105.1
C32—C33—C34	128.42 (17)	O3^iv^—Na4—H421	73.9
C33—C34—H34A	109.5	Na1^iv^—Na4—H421	119.4
C33—C34—H34B	109.5	O21^vi^—Na5—O4	96.07 (5)
H34A—C34—H34B	109.5	O21^vi^—Na5—O22^ii^	86.80 (5)
C33—C34—H34C	109.5	O4—Na5—O22^ii^	83.71 (4)
H34A—C34—H34C	109.5	O21^vi^—Na5—O51	112.08 (5)
H34B—C34—H34C	109.5	O4—Na5—O51	93.32 (4)
C33—C34—H34D	109.5	O22^ii^—Na5—O51	161.11 (5)
H34A—C34—H34D	141.1	O21^vi^—Na5—O31	178.58 (5)
H34B—C34—H34D	56.3	O4—Na5—O31	83.09 (4)
H34C—C34—H34D	56.3	O22^ii^—Na5—O31	91.97 (5)
C33—C34—H34E	109.5	O51—Na5—O31	69.14 (4)
H34A—C34—H34E	56.3	O21^vi^—Na5—O5	88.01 (4)
H34B—C34—H34E	141.1	O4—Na5—O5	162.77 (5)
H34C—C34—H34E	56.3	O22^ii^—Na5—O5	79.79 (4)
H34D—C34—H34E	109.5	O51—Na5—O5	100.67 (4)
C33—C34—H34F	109.5	O31—Na5—O5	92.47 (4)
H34A—C34—H34F	56.3	O21^vi^—Na5—Na2^vi^	45.38 (3)
H34B—C34—H34F	56.3	O4—Na5—Na2^vi^	139.98 (4)
H34C—C34—H34F	141.1	O22^ii^—Na5—Na2^vi^	83.76 (4)
H34D—C34—H34F	109.5	O51—Na5—Na2^vi^	109.59 (4)
H34E—C34—H34F	109.5	O31—Na5—Na2^vi^	135.19 (4)
C31—C35—H35A	109.5	O5—Na5—Na2^vi^	42.81 (3)
C31—C35—H35B	109.5	O21^vi^—Na5—Na3	134.47 (4)
H35A—C35—H35B	109.5	O4—Na5—Na3	47.57 (4)
C31—C35—H35C	109.5	O22^ii^—Na5—Na3	65.93 (3)
H35A—C35—H35C	109.5	O51—Na5—Na3	98.40 (4)
H35B—C35—H35C	109.5	O31—Na5—Na3	44.21 (3)
O6—C41—O5	125.44 (15)	O5—Na5—Na3	119.51 (4)
O6—C41—C4	118.26 (13)	Na2^vi^—Na5—Na3	148.72 (2)
O5—C41—C4	116.30 (14)	O21^vi^—Na5—Na2^ii^	85.72 (4)
O8—C42—O7	124.95 (15)	O4—Na5—Na2^ii^	123.06 (4)
O8—C42—C4	115.47 (13)	O22^ii^—Na5—Na2^ii^	39.43 (3)
O7—C42—C4	119.54 (13)	O51—Na5—Na2^ii^	138.34 (4)
C41—O5—Na2^vi^	119.93 (10)	O31—Na5—Na2^ii^	93.78 (3)
C41—O5—Na2^ii^	118.67 (10)	O5—Na5—Na2^ii^	40.38 (3)
Na2^vi^—O5—Na2^ii^	90.52 (5)	Na2^vi^—Na5—Na2^ii^	56.27 (2)
C41—O5—Na5	133.06 (10)	Na3—Na5—Na2^ii^	93.46 (2)
Na2^vi^—O5—Na5	89.22 (4)	O21^vi^—Na5—H31B	161.5
Na2^ii^—O5—Na5	94.76 (4)	O4—Na5—H31B	80.8
C41—O6—Na4	159.80 (11)	O22^ii^—Na5—H31B	110.8
C42—O8—Na2	134.19 (11)	O51—Na5—H31B	50.3
O3—Na1—O3^i^	180.00 (8)	O31—Na5—H31B	19.5
O3—Na1—O12^i^	90.88 (4)	O5—Na5—H31B	100.4
O3^i^—Na1—O12^i^	89.12 (4)	Na2^vi^—Na5—H31B	139.0
O3—Na1—O12	89.12 (4)	Na3—Na5—H31B	54.5
O3^i^—Na1—O12	90.88 (4)	Na2^ii^—Na5—H31B	111.4
O12^i^—Na1—O12	180.00 (5)	Na4^v^—O11—Na1	82.24 (4)
O3—Na1—O11^i^	88.82 (4)	Na4^v^—O11—H111	141.9
O3^i^—Na1—O11^i^	91.18 (4)	Na1—O11—H111	94.7
O12^i^—Na1—O11^i^	101.84 (4)	Na4^v^—O11—H11B	111.7
O12—Na1—O11^i^	78.16 (4)	Na1—O11—H11B	84.6
O3—Na1—O11	91.18 (4)	H111—O11—H11B	105.7
O3^i^—Na1—O11	88.82 (4)	Na1—O12—Na4^vii^	81.56 (4)
O12^i^—Na1—O11	78.16 (4)	Na1—O12—H121	110.1
O12—Na1—O11	101.84 (4)	Na4^vii^—O12—H121	123.7
O11^i^—Na1—O11	180.00 (7)	Na1—O12—H122	116.0
O3—Na1—Na4^vii^	56.04 (3)	Na4^vii^—O12—H122	113.2
O3^i^—Na1—Na4^vii^	123.96 (3)	H121—O12—H122	109.7
O12^i^—Na1—Na4^vii^	130.22 (3)	Na5^iii^—O21—Na2	92.39 (5)
O12—Na1—Na4^vii^	49.78 (3)	Na5^iii^—O21—H211	106.7
O11^i^—Na1—Na4^vii^	48.58 (3)	Na2—O21—H211	91.8
O11—Na1—Na4^vii^	131.42 (3)	Na5^iii^—O21—H212	146.6
O3—Na1—Na4^v^	123.96 (3)	Na2—O21—H212	95.0
O3^i^—Na1—Na4^v^	56.04 (3)	H211—O21—H212	105.5
O12^i^—Na1—Na4^v^	49.78 (3)	Na2—O22—Na5^ii^	99.04 (5)
O12—Na1—Na4^v^	130.22 (3)	Na2—O22—H221	104.5
O11^i^—Na1—Na4^v^	131.42 (3)	Na5^ii^—O22—H221	120.1
O11—Na1—Na4^v^	48.58 (3)	Na2—O22—H222	119.2
Na4^vii^—Na1—Na4^v^	180.00 (2)	Na5^ii^—O22—H222	107.5
O3—Na1—H111	88.4	H221—O22—H222	107.1
O3^i^—Na1—H111	91.6	Na3—O31—Na5	90.24 (4)
O12^i^—Na1—H111	96.9	Na3—O31—H31A	140.3
O12—Na1—H111	83.1	Na5—O31—H31A	103.5
O11^i^—Na1—H111	161.1	Na3—O31—H31B	113.4
O11—Na1—H111	18.9	Na5—O31—H31B	78.3
Na4^vii^—Na1—H111	115.8	H31A—O31—H31B	105.8
Na4^v^—Na1—H111	64.2	Na4—O41—Na4^ii^	132.60 (9)
O3—Na1—H11B	73.0	Na4—O41—H411	97.1
O3^i^—Na1—H11B	107.0	Na4^ii^—O41—H411	108.3
O12^i^—Na1—H11B	71.1	Na4—O42—H421	98.2
O12—Na1—H11B	108.9	Na4—O42—H422	105.5
O11^i^—Na1—H11B	160.1	H421—O42—H422	107.9
O11—Na1—H11B	19.9	Na5—O51—H511	134.1
Na4^vii^—Na1—H11B	121.6	Na5—O51—H512	107.4
Na4^v^—Na1—H11B	58.4	H511—O51—H512	107.0

Symmetry codes: (i) −*x*, −*y*+1, −*z*; (ii) −*x*, *y*, −*z*+1/2; (iii) *x*, −*y*, *z*+1/2; (iv) *x*, *y*−1, *z*; (v) −*x*, −*y*, −*z*; (vi) *x*, −*y*, *z*−1/2; (vii) *x*, *y*+1, *z*; (viii) −*x*, −*y*, −*z*+1.

### Hydrogen-bond geometry (Å, °)

**Table:**

*D*—H···*A*	*D*—H	H···*A*	*D*···*A*	*D*—H···*A*
O11—H111···O2^ii^	0.86	1.97	2.7404 (16)	149
O11—H11B···O4	0.85	1.88	2.7067 (16)	165
O12—H121···O1^ii^	0.84	1.90	2.7391 (17)	174
O21—H211···O6^ii^	0.85	1.92	2.7566 (17)	167
O21—H212···O7	0.85	1.97	2.8007 (16)	165
O22—H221···O31	0.85	2.10	2.8381 (17)	145
O22—H222···O11^ii^	0.85	2.31	2.9707 (16)	135
O22—H222···O6^iii^	0.85	2.45	3.0532 (18)	128
O31—H31A···O8	0.85	1.87	2.7128 (16)	169
O31—H31B···O51	0.84	2.09	2.8085 (17)	142
O41—H411···O7	0.84	2.27	3.1061 (16)	172
O42—H421···O1^iv^	0.85	1.92	2.7582 (17)	175
O42—H422···O7	0.85	2.03	2.8639 (17)	168
O51—H511···N4	0.86	1.99	2.8424 (18)	171
O51—H512···N2	0.85	1.94	2.7799 (18)	169

Symmetry codes: (ii) −*x*, *y*, −*z*+1/2; (iii) *x*, −*y*, *z*+1/2; (iv) *x*, *y*−1, *z*.
